# Recent progress on field-effect transistor-based biosensors: device perspective

**DOI:** 10.3762/bjnano.15.80

**Published:** 2024-08-06

**Authors:** Billel Smaani, Fares Nafa, Mohamed Salah Benlatrech, Ismahan Mahdi, Hamza Akroum, Mohamed walid Azizi, Khaled Harrar, Sayan Kanungo

**Affiliations:** 1 Abdelhafid Boussouf University Centre of Mila, Mila, Algeria; 2 University of Jijel, Automation Department, Jijel, Algeria; 3 Laboratoire de Recherche Electrification des Entreprises Industrilles (LREEI), Faculté des Hydrocarbures et de la Chimie, Université M’Hamed Bougara Boumerdes, Algeria; 4 LIST Laboratory, University M’Hamed Bougara, Boumerdes, Algeria; 5 Department of Electrical and Electronics Engineering Birla Institute of Technology and Science Pilani, Hyderabad, India

**Keywords:** biomolecule detection, biosensors, charge modulation, dielectric modulation, field-effect transistor

## Abstract

Over the last few decades, field-effect transistor (FET)-based biosensors have demonstrated great potential across various industries, including medical, food, agriculture, environmental, and military sectors. These biosensors leverage the electrical properties of transistors to detect a wide range of biomolecules, such as proteins, DNA, and antibodies. This article presents a comprehensive review of advancements in the architectures of FET-based biosensors aiming to enhance device performance in terms of sensitivity, detection time, and selectivity. The review encompasses an overview of emerging FET-based biosensors and useful guidelines to reach the best device dimensions, favorable design, and realization of FET-based biosensors. Consequently, it furnishes researchers with a detailed perspective on design considerations and applications for future generations of FET-based biosensors. Finally, this article proposes intriguing avenues for further research on the topology of FET-based biosensors.

## Review

### 1 Introduction

**Figure 1:**
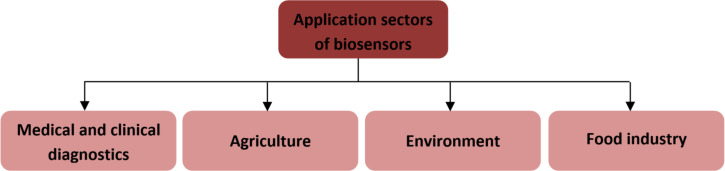
Schematic showing main sectors of biosensor applications.

In recent years, biosensor devices have gained significant importance across various domains, including the medical field, environmental monitoring, and the agricultural sector (Figure 1) [1–2]. In this context, biosensors have found widespread application in industries, particularly for food quality control and safety [3]. They are employed in agriculture [4] during crop cultivation as well as in food processing. Quality control is essential to ensure wholesome food production with an extended lifespan [5]. Biosensors have been implemented in at-line and on-line quality sensors [6], enabling quality classification, automation, and reducing costs and time. Biosensors have also been designed to detect chemical and biological substances in food items, which might be contaminants or undesirable elements [5,7–9]. The presence of chemicals is often considered an environmental problem as chemical species contaminate water bodies. Therefore, the detection of harmful pollutants in the environment is a critical issue. Numerous works have reported on the application of biosensors for environmental monitoring, especially those based on optical or electrochemical transduction platforms [10–13].

Moreover, biosensors have been extensively utilized in the field of medical sciences and clinical diagnostics [14]. They have been employed in cancer diagnosis [15], cardiovascular studies [16], and diabetes monitoring [17]. The application of biosensors in cancer diagnosis and therapy is very important due to the widespread frequency of the disease, high mortality rate, and recurrence after treatment. In addition, biosensors are applied to monitor blood glucose levels in diabetics, identify infections, and track cancer growth [14]. The development of biosensor technologies for cancer screening is crucial and beneficial [18]. Additionally, biosensors have been used to detect SARS-CoV-2, which causes COVID-19-related severe respiratory distress [19–20]. For the accurate detection of COVID-19 RNA [21–22], proteins [23–24], and virus particles [25–26], various methods have been proposed, such as CRISPR systems [27–28], surface-enhanced Raman spectroscopy [29–30], microfluidic-coupled biochip [31], electrochemical [32], and field-effect transistor (FET)-based biosensors [33].

Biosensors offer several distinct benefits for virus recognition, including higher selectivity through improved target receptors and good sensitivity detection via a label-free procedure, real-time electrical signal in situ amplification, and cost-effective mass production, achieved through microelectronic manufacturing processes and a small size for portable point-of-care testing [34–35]. Additionally, the application of biosensors for accurate detection of viruses [25], cancer [15], proteins [36], DNA, glucose [17], and nucleic acids has been strongly developed [37].

On the other hand, specific biomolecule classifications by microbiologists has led to the realization and development of different biosensors, significantly increasing their use in daily life [38]. The first ion-sensitive field-effect transistor (IS FETs) biosensor combined the metal–oxide–semiconductor (MOS) structure with glass electrodes for measuring ion activities in electrochemical and biological environments [39]. Subsequently, hydrogen-sensitive MOSFET technology rapidly emerged [40–41]. However, all these FETs were highly bulky and required more space. Nakamoto et al. [42] later devised a biosensor with a novel fabrication method aligned well with the complementary metal–oxide–semiconductor (CMOS) fabrication process.

Usually, biosensors convert biological characteristics of the target biomolecules into measurable and quantifiable electrical signals [43]. In this sense, various types of biosensors have been designed using electrical [44], thermal [45], and optical signals [46]. Among these, FET-based biosensors have garnered significant attention from researchers due to their desirable properties and advantages, including low cost, label-free operation, high sensitivity, robustness, low power consumption, and a straightforward fabrication process based on CMOS technology [47–48].

**Figure 2:**
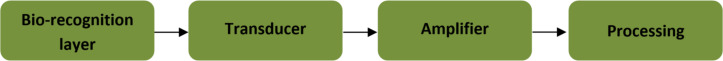
Schematic showing the organization of FET-based biosensors.

**Figure 3:**
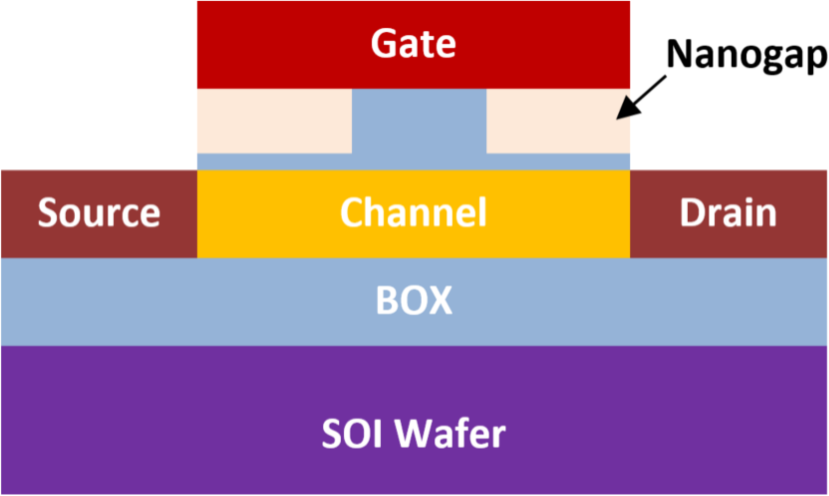
Schematic showing the general structure of DM FET-based biosensors with two nanogaps.

**Figure 4:**
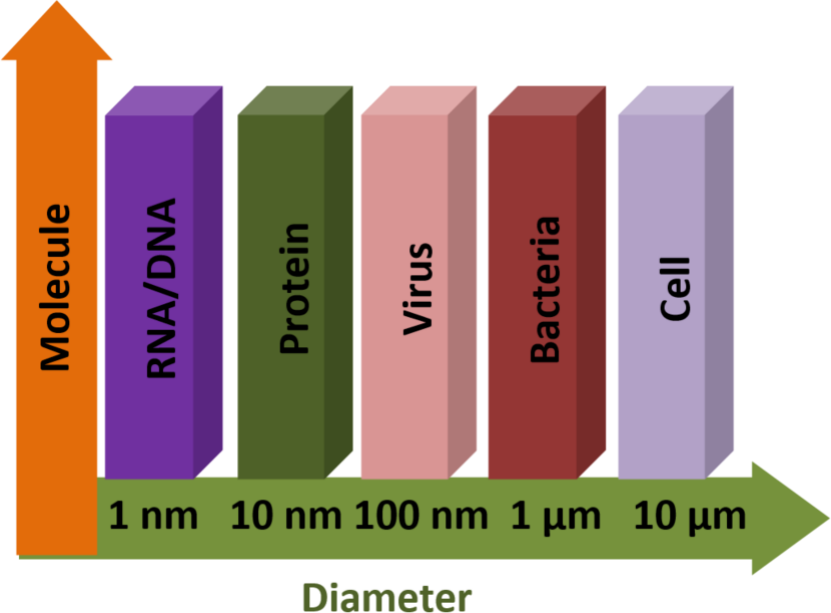
Adequate diameter for detecting different molecule types.

A field-effect transistor-based biosensor comprises a biorecognition layer, a transducer, and an amplifier (Figure 2). It typically consists of a semiconductor channel and three terminal electrodes named drain, source, and gate [37,49]. The biorecognition layer selectively binds the target biomolecule (analyte), such as enzymes, antibodies, and proteins in a heterogeneous environment. Additionally, the amplifier increases the signal that is produced by the transducer element [50]. In this sense, the transducer is responsible for converting the interaction that occurs between the biorecognition component and the analyte into a signal. Also, an immobilized biological sensing membrane is accommodated between the contact of the metal gate and the insulator part. The carrier concentration in the body channel might change as a result of an accumulation, depletion, or inversion process when an external voltage is applied [51]. This results in the formation of a band bending between the carriers. However, when the gate bias that is supplied is greater than the threshold voltage of the device, the type of the channel is inverted which permits the current to flow and turns on the transistor [52]. The FET-based biosensors consist of two dissimilar transduction mechanisms. The first transduction mechanism is known as charge modulation, in which charged biomolecule species bind to the surface of the gate insulator and modify the charge density of the channel surface, and thus the surface conductivity by Coulomb interaction. This acts as a gating mechanism, and concurrently shifts the threshold voltage and modifies the drain current via channel conductivity modulation. The difference between the current (threshold voltage) in the presence of biomolecules and the current without biomolecules is used to define the current sensitivity. The second transduction mechanism is known as dielectric modulation, where a nanoscale gap cavity is introduced in either the gate metal or gate insulator region. The biomolecules are immobilized in this cavity region functionalized with bioreceptor elements, where the presence of the specific target biomolecule alters the effective dielectric constant inside the cavity resulting in a gating effect modulation and subsequent alteration in channel conductivity. This leads to a change in both threshold voltage and drain current, manifesting the presence of target biomolecules in the cavity, and the corresponding sensitivity is defined similarly to charge modulated transduction [4]. The dielectrically modulated (DM) FET is considered one of the most promising variants and approaches to field-effect transistor-based biosensors due to its unique ability of detecting both charged and uncharged biological species through charge modulation and dielectric modulation (Figure 3) [53–54]. The DM field-effect transistor structures include a nanogap cavity either in the metal gate or in the insulator gate region, within which the target biomolecules can conjugate and modify the effective gate capacitance and the effective gate channel electrostatic coupling [55]. The variation of capacitance depends on the intrinsic charge density and the dielectric constant of the target biomolecule [48]. The cavity specification and thereby the sensor device design is also related to the diameter of targeting species, which varied from micro- to nanoscale (Figure 4).

#### 1.1 Motivation of this review

The development of advanced bioelectronic technologies involves exploration of novel material systems as well as emerging device architectures. However, an accurate assessment of different device architectures and their design criteria requires a systematic modeling of the electrical characteristics of the device.

In this context, due to diversities in the transduction and applications of FET-biosensors, the primary design improvisation involves novel device architectures for FET dedicated to biosensing applications. Therefore, an extensive and systematic review on emerging structures of FET-based biosensors is important for the design of future nanoscale FET-biosensors for different applications. There are a few reviews published regarding FET-based biosensors [56–59]. Most of these works are focused on the materials-based performance optimization of FET-based sensors, and a few reviews of FET-based biosensors report on the analysis and development of nanostructured materials for those biosensors [37]. However, to the best of our knowledge, none of the reported reviews have proposed a comprehensive overview on various structures for the design of FET-based biosensors. Therefore, this current article provides a comprehensive review of FET-based biosensor architectures. More specifically, apart from conventional MOSFET, the development of emerging non-CMOS devices including biosensors based on tunnel-field effect transistors (TFETs) and negative-capacitance field-effect transistors (NCFETs) has also been summarized.

#### 1.2 Organization of this review

This review is organized as follows: In the first section, we provide an introduction to FET-based biosensors, where we discuss biosensor technology and primary applications. The second section presents the latest emerging structures of FET-based biosensors. The third section presents a comparative analysis between the latest FET-based biosensors. The fourth section presents a summary and future research works. The final section concludes the manuscript.

### 2 Emerging FET-based biosensors

#### 2.1 Three-dimensional FET-based biosensors

**Figure 5:**
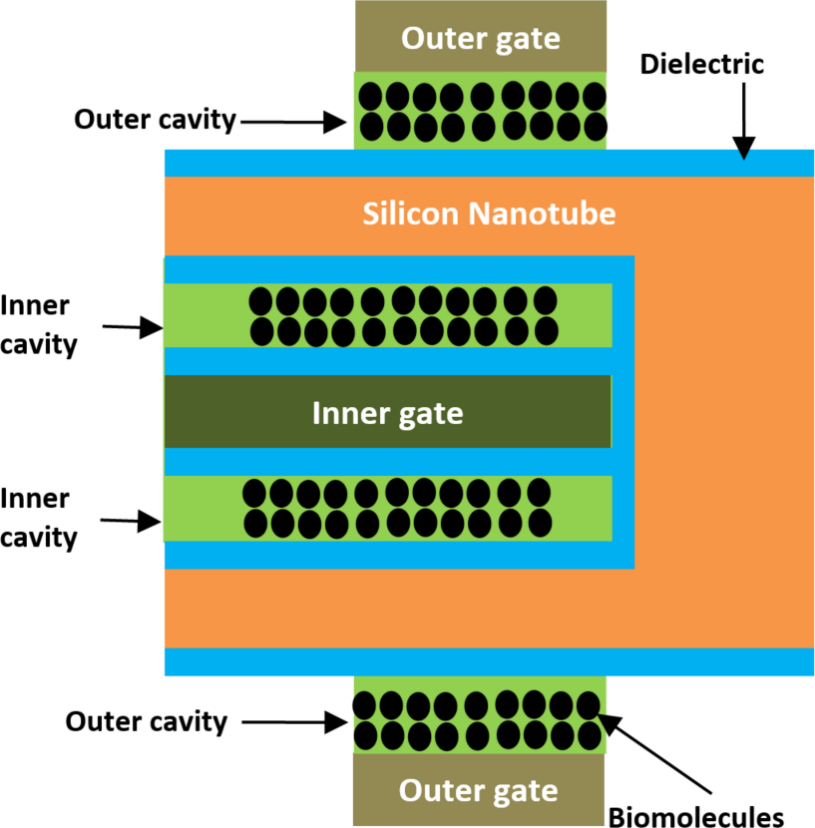
Schematic showing the cross-sectional representation of a HG NT JL FET-based biosensor.

**2.1.1 Nanotube FET-based biosensors.** Tayal et al. [60] introduced a heterogate nanotube junctionless (HG NT JL) FET-based biosensor structure. The gate-all-around (GAA) structure is implemented for better electrostatic integrity regarding the dielectric and charge modulation [61]. Figure 5 shows the 2D representation of the structure of a silicon HG NT JL FET-based biosensor. In this case, the full architecture of a nanotube FET has been used to design high performance biosensors. There are two types of gate cavity: the inner gate cavity and the outer gate cavity, as shown in Figure 5. The inner gate cavity length and outer gate cavity length are equal to 150 nm and 100 nm, respectively. A 1 nm thick insulator is implemented at the top of the outer and inner cavity regions to ensure reliable isolation between the active region and the biomolecules [62]. The thickness of the inner and outer gate cavity regions is set to 10 nm [63]. Therefore, the physical properties of the biosensor have been analyzed via source-channel junction electrostatics and thermionic emission. The effect of applying biasing conditions on the efficiency of the transduction is also investigated. It has been demonstrated that the HG NT JL FET-based biosensor architecture enhances sensing performance for different charged and neutral biomolecules through the nanotube-gate concept [60].

**Figure 6:**
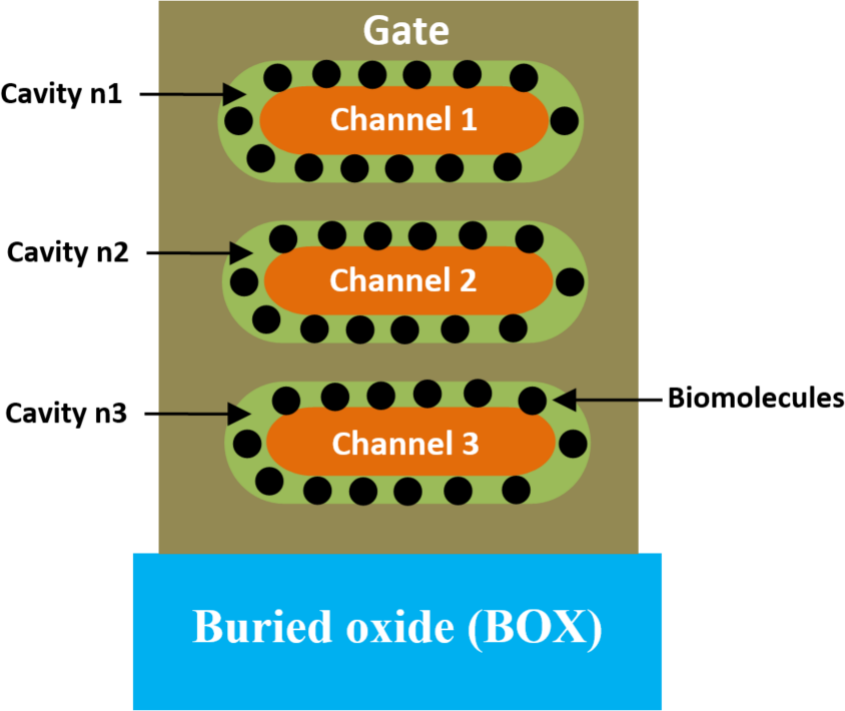
Schematic showing the cross-section (2D) of a VS NS FET-based biosensor.

**2.1.2 Nanosheet-based biosensors.** Li et al. [64] proposed a vertically stacked gate-all-around nanosheet (VS NS FET)-based biosensor. Figure 6 shows the 2D representation of silicon VS NS FET-based biosensor designed on a buried oxide (BOX). In this structure, three similar nanocavity regions were created between the three channels and the metal gate to immobilize targeted biomolecules, as shown in Figure 6. The gate metal around the three nanocavity regions was added for the best electrostatic control. Additionally, a spacer near the source region was removed to facilitate the introduction of biomolecules into the cavity. The fabrication process flow of the proposed VS NS FET has been detailed by Ryu et al. [65] and Tsai et al. [66], and the formation of the cavity has been demonstrated by Buitrago et al. [67]. Due to the three stacked channels of the VS NS FET, both the on-current and output characteristics were significantly improved [68–70]. Furthermore, since the channel width can be flexibly adjusted through layout design, there is a broader enhancement space for biosensor design [71]. Moreover, owing to the larger channel surface and wider channel, biomolecules can be easily immobilized in the nanocavity. Consequently, the VS NS FET-based biosensor provides higher sensitivity.

**Figure 7:**
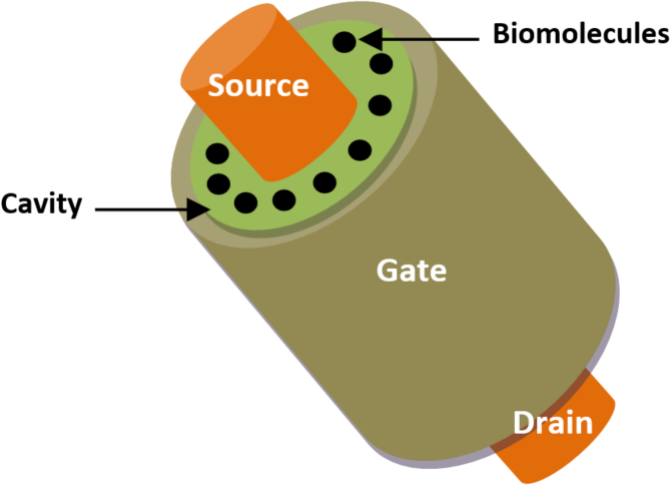
Schematic showing the structure of the 3D SRG JL FET-based biosensor.

**2.1.3 Surrounding-gate FET-based biosensors.** Pratap et al. [72] proposed a nanogap cavity embedded surrounding-gate junctionless (SRG JL) FET-based biosensor for the electrochemical detection of different types of charged and neutral biomolecule species, such as APTES, uricase, ChOx, proteins, and streptavidin. Figure 7 shows the 3D representation of an SRG JL FET-based biosensor. One type of doping concentration was added to the silicon channel, source, and drain region. A surrounding cavity was created between the oxide and the gate metal. This structure utilizes a silicon-based substrate with SiO_2_ as an interface dielectric layer. The body channel, source and drain regions were homogeneously doped. The surrounding-gate architecture improves the sensitivity of the biosensor via the bulk conduction process and reduces short-channel effects. Additionally, it has been demonstrated that the SRG JL concept provides better sensitivity and a simpler fabrication process than inversion-mode device-based biosensors, considering a single type of body doping concentration [72].

Another junctionless (JL) surrounding-gate stack FET-based biosensor concept has been analyzed and proposed by Chakraborty et al. [73]. This structure uses two surrounding nanogap cavities separated by HfO_2_ as a high-*k* dielectric material and SiO_2_ as an interface layer. It has been reported that this structure offers higher sensitivity compared to that of the dual-material JL MOSFET-biosensor proposed by Ahangari et al. [74] and the double-gate JL MOSFET biosensor proposed by Narang et al. [75].

**Figure 8:**
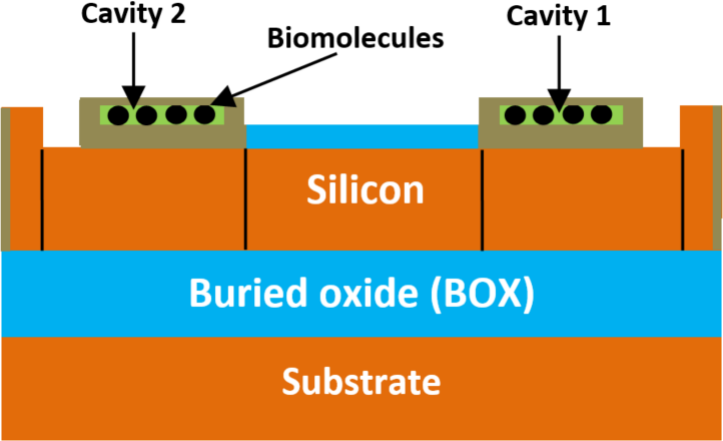
Schematic showing the cutting-plane view of a FinFET-based biosensor.

**2.1.4 FinFET-based biosensors.** A FinFET-based biosensor structure has been designed and proposed by Kesherwani et al. [76] with a 40 nm channel length for label-free applications. Figure 8 shows the 2D representation of a FinFET-based biosensor structure. In this FET-biosensor architecture, two cavities with a 5 nm thickness are introduced on both sides of the fin to form a high-performance sensing surface for various types of biomolecules. Additionally, a nanocavity was created between the gate and the source–drain region to immobilize the biomolecules. The biomolecule species are introduced inside the nanocavity for immobilization [76]. The ability of the proposed FinFET-biosensor to detect various biomolecule species has been examined in terms of sensitivity. However, the maximum reported biosensor sensitivity is equal to 1.55 for a constant dielectric equal to 8.

Various circuit architectures combining both sensing and signal readout functions have been investigated by Rigante et al. [77]. In this context, the FinFET device was implemented in both the metal gate and the sensor device. Moreover, the FinFET structure with a hybrid partially gated design is reported as an exceptional device allowing digital gates with biosensing integration. Also, a hardware description language (Verilog-A) has been used for the modeling of each device structure to be incorporated into various system designs via electronic design automation simulations (EDAS) [77–78]. Furthermore, an amplifier with common-source architecture has been incorporated into a simple analog circuit for the amplification of the threshold-voltage change, leading to the measurement of pH change at the level of the biosensor surface. Thus, two different circuits for signal conditioning and biosensing have been implemented, such as the pseudo-differential amplifier and ring oscillator circuits. It has been demonstrated that the FinFET-based sensing amplifier exhibits a readout sensitivity that is at least ten times higher than that of individually addressable single sensors, where the sensitivity parameter is limited by the fundamental Nernst equation. It has also been reported that the signal improvement results from the designed circuit, and the sensitivity limit has been addressed by means of the back gate architecture [79–80], or the structure of double-side gates [81–82].

**Figure 9:**
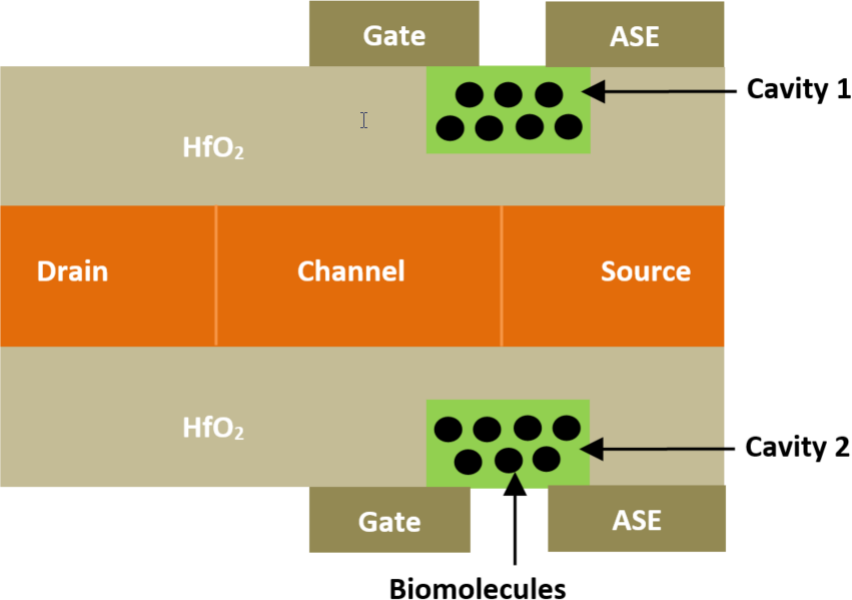
Schematic showing the cross-section of an NW TFET-based biosensor with an additional electrode (ASE).

**2.1.5 Nanowire FET-based biosensors.** As planar FET devices face several challenges, such as the weak controllability of the gate electrode through the channel, alternative solutions have been explored. The concept of nanowire tunnel FETs (NW TFET) was proposed by Soni et al. [83] for biosensing applications. Figure 9 shows the 2D representation of NW TFET-based biosensors. In this structure, a double gate and two similar nanocavity regions were created to immobilize targeted biomolecules. A high-*k* material was implemented between the gate–metal and channel–source–drain regions. Besides, an additional electrode (ASE) was placed around the cavity and the source region in the oxide zone below the gate, and extended towards the source–oxide region, as shown in Figure 9. So, by using the source electrode, additional holes were created and accumulated on the surface of the source region, forming a plasma layer of holes. An abrupt junction at the channel/source was created to facilitate the entry of biomolecules into the cavity. Thus, significant variations in electrostatic properties were observed due to the distinct characteristics of the biomolecules, leading to improved sensitivity (≈10^8^) and increased sensing speed through the extended cavity in the oxide–source region of the proposed biosensor structure.

Furthermore, the fabrication of a silicon NW TFET-based biosensor has been reported by Gao et al. [84], targeting high sensitivity, versatility, and reliability for point-of-care diagnostic systems. This device was realized using a top-to-down approach with an anisotropic and cost-effective self-stop etching method [85–86]. A novel CMOS anisotropic technique was implemented for the etching process, combining classical optical and electron beam lithography with anisotropic wet etching via the tetramethylammonium hydroxide method. This structure offers anti-interference and strong capability, demonstrating inherent ambipolarity through CYFRA21-1 and pH sensing. The device proposed by Gao et al. [84] provides enhanced operational conditions compared to classical FET-based biosensors, with a low limit of quantification and detection.

A real-time simulation of a highly sensitive specific antigen biosensor was also performed by Gao et al. [87] using silicon NW FET-based CMOS technology. In this work, both P- and N-type NW arrays were designed and incorporated into one chip using CMOS technology, combined with optical lithography and an anisotropic self-stop etching method [88–89]. The incorporated P- and N-type NWs showed complementary electrical responses upon prostate-specific antigen binding, providing a unique means of internal command for biosensing signal verification. It was demonstrated that this biosensor structure is reliable, accurate, and effective at concentrations as low as 1 fg·mL^−1^, making it suitable for prostate cancer and clinical diagnosis applications. Additionally, this structure is low cost and exhibits a good ability for the detection of biomolecule species.

Wenga et al. [90] designed a step-gate polysilicon FET-based biosensor for the detection of DNA. An emerging step-gate polycrystalline silicon NW FET-based biosensor was fabricated to enable highly sensitive electrical biosensing for DNA hybridization detection with a low-cost and simple fabrication process. The polysilicon NW FET (PSi-NW) is based on a silicon NW transistor with CMOS technology, implemented in highly sensitive biosensors for the detection of biological species. The PSi-NW is synthesized using the sidewall spacer formation technique and designed with a five-mask process with a maximum temperature of 600 °C, featuring N-type devices with different parallel polysilicon channels using the side-wall spacer technique. It has been reported that the developed step-gate PSi-NW FET-based biosensor offers a high surface-to-volume ratio and low-cost devices, allowing better and direct sensitive recognition of various biological species.

Another silicon NW-based biosensor concept was reported by Buitrago et al. [91]. This NW structure uses a vertically stacked architecture and full-depleted body channels for better array concentration. The classical top-down clean room method was employed for the fabrication of the NW structure [92]. Moreover, this device is gated by a back gate and one or two platinum side gates via a liquid which has been characterized for possible implementation in reliable sensing applications. This biosensor uses HfO_2_ as a high-*k* gate dielectric material and silicon-on-insulator substrates with low-doped device layers and small nanowire diameters, achieving a fully depleted mechanism and allowing better surface-to-volume ratios and higher sensitivity applications.

#### 2.2 Two-dimensional FET-based biosensors

**Figure 10:**
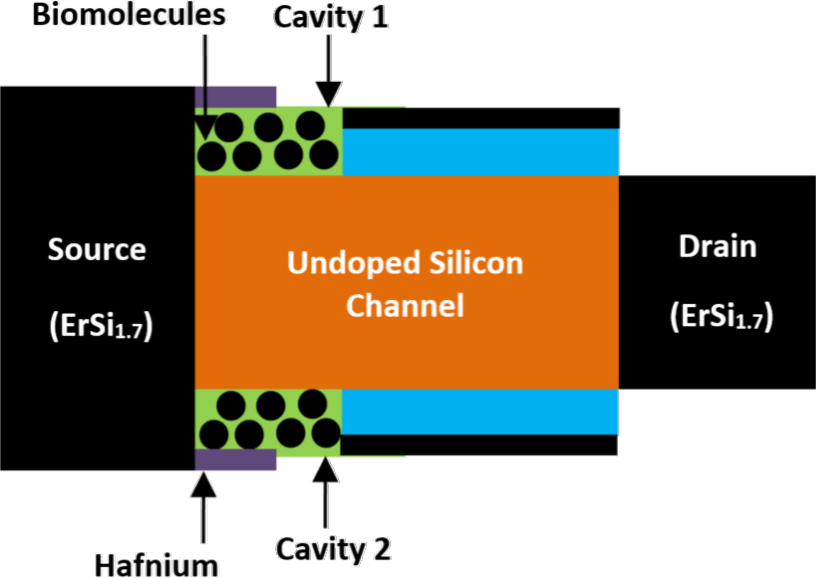
Schematic showing the view of an SE SB FET-based biosensor.

**2.2.1 Source-engineered Schottky barrier FET-based biosensors.** A Schottky barrier (SE SB) FET-based biosensor, engineered with a charge plasma source, has been proposed and simulated by Hafiz et al. [93], targeting biosensing applications. Figure 10 shows the innovative structure of SE SB FET-based biosensor which utilizes a hafnium material with a work function of 3.8 eV for source extensions and erbium silicide (ErSi1.7) materials for the drain and source regions. Two separated horizontal L-shaped nanocavity regions were implemented between the undoped silicon channel and the hafinium material. The oxide beneath the source extensions was precisely etched to create separated nanocavity regions for enhancing the detection of biomolecule species. It has been reported that the SE SB FET-based biosensor structure offers a significant improvement in sensitivity at low-temperature values. Additionally, the SE SB FET-based biosensor provides enhanced sensing capability for the detection of both charged and neutral biomolecules compared to classical dielectric-modulated FET-based biosensors [93].

**Figure 11:**
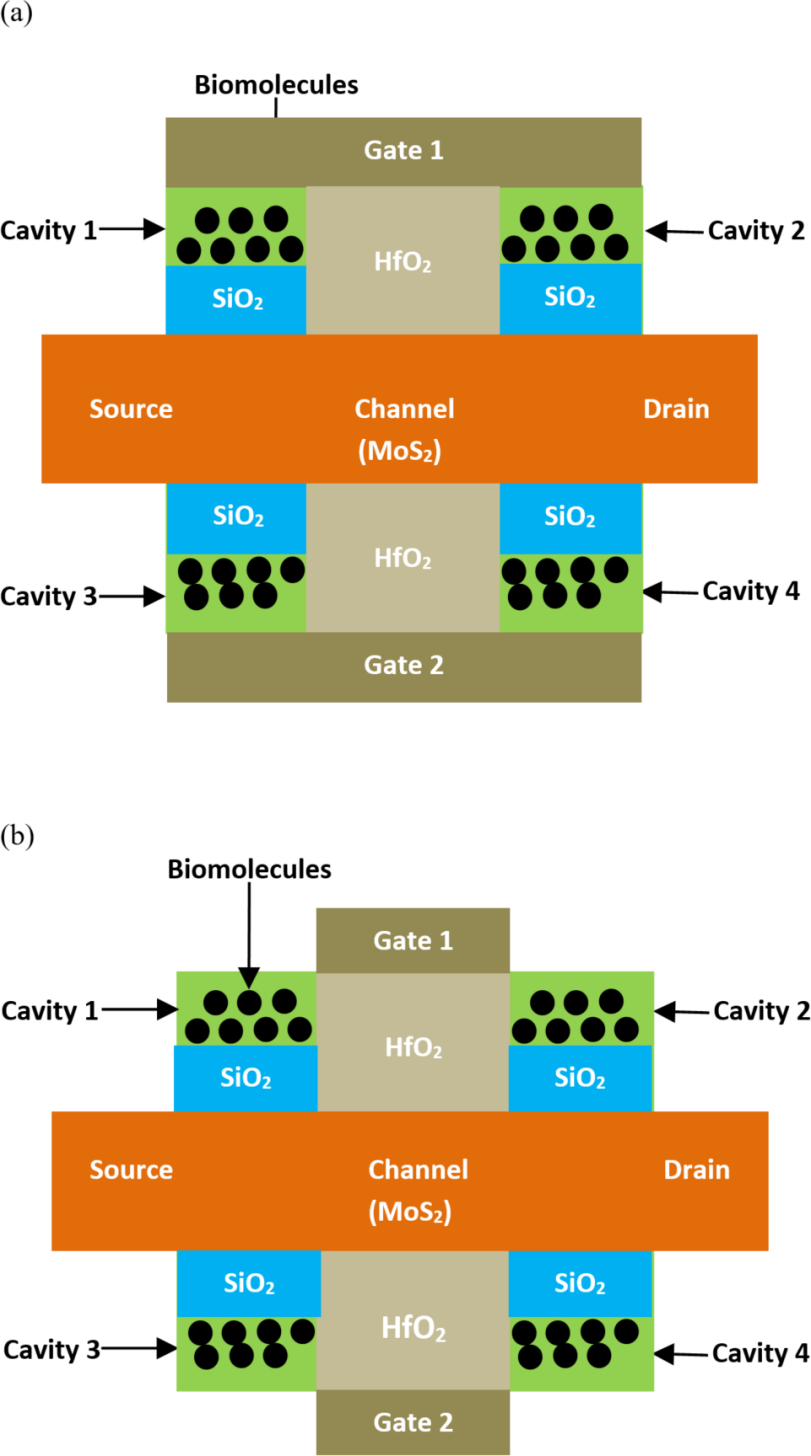
Schematic showing the structure of TMD FET-based biosensors without a gate above the nanocavity (a) and with a gate above the nanocavity (b).

**2.2.2 Transition metal dichalcogenides-based biosensors.** Kumari et al. [94] investigated a double-gate biosensor based on transition metal dichalcogenides (TMD FET) for label-free biosensing. In this study, two different structures were considered for the TMD FET biosensor: (1) a TMD FET biosensor without a gate above the nanocavity (Figure 11a), and (2) a TMD FET biosensor with a gate above the nanocavity (Figure 11b) using the DM approach to include biomolecule species. This structure utilizes MoS_2_ in the body channel region and HfO_2_ as a high-*k* dielectric placed between the electrode gates and the body channel. It has been indicated that the sensitivity parameter of the designed biosensor is almost 100% higher in the case of the TMD FET biosensor without a gate above the nanocavity for biomolecules with a dielectric constant equal to 12, compared to the structure of the TMD FET biosensor with a gate above the nanocavity for fully-filled cavities.

**2.2.3 Split-gate junctionless FET-based biosensors.** A dielectric-modulated split-gate junctionless (SPG JL) FET-based biosensor was introduced by Singh et al. [95] for label-free detection of various biomolecule types (neutral and charged).

**Figure 12:**
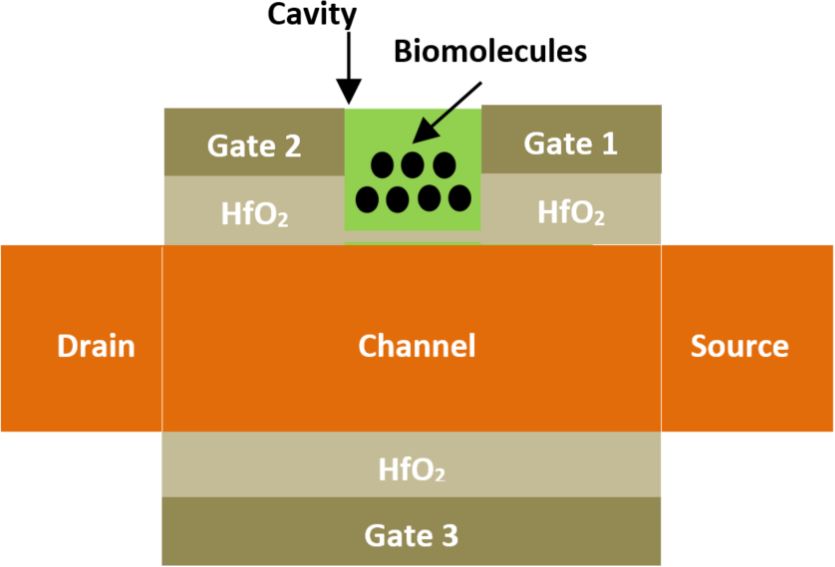
Schematic showing the structure of an SPG JL FET-based biosensor.

Figure 12 shows the structure of a silicon SPG JL FET-based biosensor. This structure utilizes an N^+^ doping concentration in the source/channel/drain regions, three electrode gates (1, 2, and 3), and a high-*k* dielectric material (HfO_2_) between the different regions of the channel and electrode gates, as shown in Figure 12. The nanocavity has been implemented between gate 1 and gate 2. Here, the cavity length is equal to the length of the gate underlap region. The channel length, the thickness of HfO_2_, the thickness of SiO_2_, and the thickness of silicon are 225 nm, 10 nm, 1 nm, and 10 nm, respectively.

**Figure 13:**
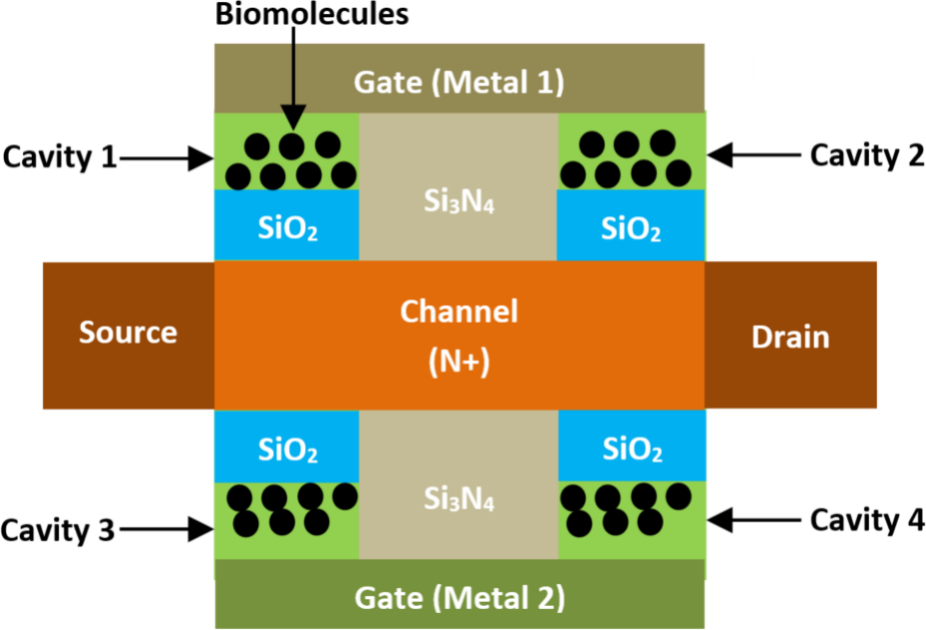
Schematic showing the structure of a DC DG JL MOSFET-based biosensor.

**2.2.4 Dual-cavity JL MOSFET-based biosensors.** Jana et al. [96] reported a dual-cavity double-gate junctionless (DC DG JL) MOSFET-biosensor targeting real-time biosensing applications. Figure 13 shows the schematic view of a DC DG JL MOSFET-based biosensor. In this structure, double cavities were created in each side of the device between the gate electrodes and the dielectric SiO_2_ layer, and also separated with a Si_3_N_4_ dielectric region targeting high-sensing performance. Also, two different metals were considered for the electrode gates. A uniform doping concentration was considered for channel, source, and drain regions for simplifying the device fabrication. The current sensitivity, intrinsic delay, and power consumption of this biosensor were investigated. For a dielectric constant equal to 12, the proposed structure provides a power dissipation of 2.8 pW and a maximum current sensitivity of 104%. Moreover, it was demonstrated that the dual-cavity topology offers a higher efficacy than the single cavity in terms of power consumption and current sensitivity [96]. Furthermore, the power dissipation is lower and the delay is much larger. Additionally, power dissipation is different for charged and neutral biomolecules. Furthermore, the transient response of the proposed biosensor adds a diverse aspect when analyzing the delay of the biosensor and after the immobilization of biomolecules in the cavity.

**2.2.5 Charge plasma four-gated MOSFET-based biosensors.** Chanda et al. [97] designed a charge plasma four-gated (CP FG) MOSFET-based biosensor structure to enhance the detection of various types of biomolecules. The concept of charge plasma has been developed and implemented using appropriate metal work-function electrodes [98–99]. This structure is akin to the classical double-gate device, with the exception that the mid-region between the gates is etched out to capture biomolecules. The device reported by Chanda et al. can be realized by following the techniques described by Ahm et al. [81]. Additionally, the proposed structure has a gate length of 300 nm, an underlap length between the gates of 200 nm, a channel thickness of 25 nm, a thickness of the front and back gate oxide of 10 nm, an oxide thickness layer at the mid-region of 1 nm, and a channel doping concentration of 10^16^ cm^−3^. Moreover, significant improvements on the on-current state and on the threshold voltage of the device are observed when charged biomolecules are immobilized. It has also been indicated that the CP FG MOSFET-based biosensor provides high sensitivity and a low thermal budgeting scheme. Therefore, this structure could be a promising candidate to replace classical FET-based biosensors due to its low thermal budgeting and compatibility with the fabrication processes of CMOS technology [97].

**Figure 14:**
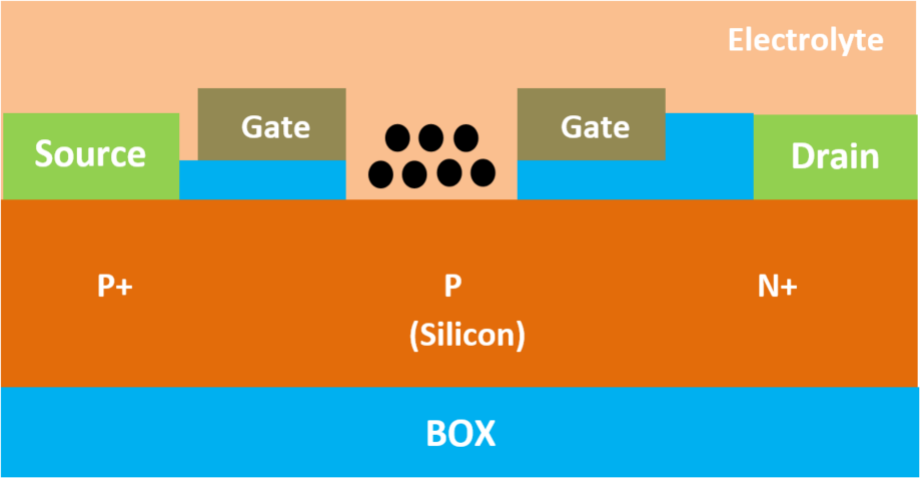
Schematic showing the structure of a UII MOS-based biosensor.

**2.2.6 Underlap impact-ionization MOS-based biosensors.** Kannan et al. [100] proposed an underlap impact-ionization MOS (UII MOS) device structure for label-free biosensing systems. Figure 14 shows the architecture of a UII MOS-based biosensor. This structure uses an underlap concept in the middle of the gate architecture, creating a self-aligned gate-first process [101–102]. Therefore, it reduces the changes in the underlap length and eliminates the need for a gate alignment step required in a gate-last process [103]. This type of biosensor is also compatible with the conventional process of CMOS technology and exhibits individual addressing in a biosensor array through the gate electrode [101–102]. It has also been demonstrated that the UII MOS-based biosensor offers a higher and better sensitivity value (≈10^7^) even in a humid environment, as compared with the classical underlap FET-biosensor for biomolecule species detection [104].

**2.2.7 Inverter and ring oscillator-based biosensors.** Kanungo et al. developed an inverter-based strategy for dielectric-modulated biosensing applications [105]. In this case, a bioring oscillator was incorporated for the detection of biomolecules. Thus, the biomolecule species are detected through the oscillation frequency amplification with conjugation. Therefore, a DM fringing FET-based transducer was implemented as the pull-up and pull-down elements for this bioinverter. Also, different bioinverter structures and configurations have been reported for adequate detection of uncharged and charged biomolecule species. It has been indicated that the sensor/sensor and “P”- type FET-sensor configurations are the most suitable candidates for the detection of uncharged and charged biomolecule species. However, for the realization of bioring oscillators, the sensor/sensor configuration is suitable for the detection of uncharged biomolecules, whereas the P-type FET-sensor configuration is adequate for the detection of positively charged and the sensor N-type configuration is suitable for negatively charged biomolecules.

**2.2.8 MoS2 FET-based biosensors.** Nam et al. [106] identified two different physical principles for the accurate operation of MoS_2_ FET-based biosensors based on the positions of antibody functionalization. When antibodies are immobilized at the level of the insulated layer covering the MoS_2_ device, antibody–antigen binding events mostly change the threshold voltage of the device, which can be described by the classical capacitor-model approach. Moreover, if the antibody particles are grafted at the level of the MoS_2_ body channel, the binding events mainly modulate the on-state transconductance of the device, credited to the antigen-induced disordered potential in the MoS_2_ body channel. This physics study of the biosensor device simplifies the biosensor architecture for accurate femtomolar detection and quantification of various biomolecule species.

**2.2.9 Vertical-strained impact-ionization MOSFET-based biosensors.** Saad et al. [107] reported an equivalent electrical circuit model dedicated to vertical-strained impact-ionization (VSR II) MOSFET, which can be used for biosensing applications. The proposed structure is designed to reduce the supply voltage [108].

When the strained layer (SiGe) is implemented in the framework level, the threshold and supply voltage are considerably reduced, as well as the leakage current and subthreshold. Therefore, the VSR II MOSFET devices are promising candidates for high-sensitive biosensor applications.

**2.2.10 Extended-gate FET-based biosensors.** Guan et al. [109] designed an off-chip extended-gate (EG) FET-based biosensor with better sensitivity and non-enzymatic targeting detection of uric acid in serum and urine. In this sense, the authors developed an enzymatic potentiometric technique that can be employed for the accurate determination of uric acid density in human urine and serum. In this context, the detection of uric acid is achieved by the measurements of interfacial potential by means of the extended-gate structure combined with ferrocenyl–alkanethiol-modified gold electrodes [110]. This structure uses reusable back-end detection parts and disposable front-end biosensing chip parts. A quasi-reference electrode is made using Ag/AgCl. The front-end biosensing parts are made using gold electrode material and manufactured by a lithography process, liftoff technique, and metal evaporation process. It has been indicated that the proposed EG FET-based biosensors exhibit super selectivity and high sensitivity for a reliable detection of uric acid in human urine and serum.

**Figure 15:**
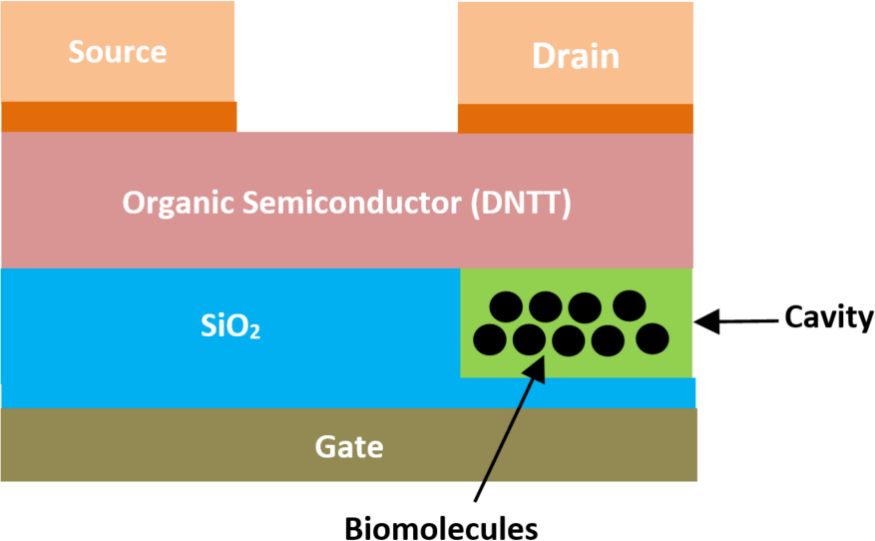
Schematic showing the structure of a BE TC OTFT-based biosensor.

**2.2.11 Organic TFET-based biosensors.** Jain et al. [111] proposed a concept of bilayer electrodes for top-contact organic tunnel FET (BE TC OTFT)-based biosensors aiming for enhanced detection of charged and neutral biomolecules. Figure 15 shows the schematic view of a BE TC OTFT-based biosensor. In this structure, one nanocavity was created below the organic semiconductor channel and above the electrode gate for better sensitivity and for advanced flexible biochip applications. Dinaphtho, thieno, and thiophene (DNTT) materials were implemented in the channel, and a bilayer of gold and TiO_2_ was used for the drain and source electrodes [112]. The body channel length is equal to 1 µm, and the cavity region thickness varied from 5 to 10 nm. It was demonstrated that BE TC OTFT is four times more sensitive compared to the metal-trench dielectric-modulated organic tunnel FET [113]. It was also indicated that this biosensor offers good flexibility and a simple, low-cost fabrication process.

**2.2.12 Pocket-doped TFET-based biosensors.** An emerging pocket-doped tunnel (PKD) FET-based biosensor has been designed for biosensing applications by Rashid et al. [114].

**Figure 16:**
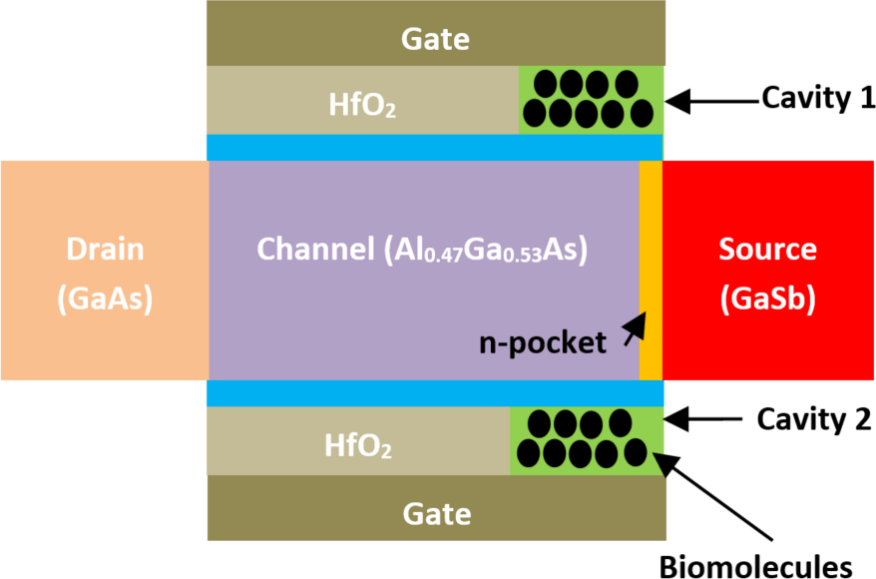
Schematic showing the structure of a PKD TFET-based biosensor.

Figure 16 presents the organization of PKD FET-based biosensor. This structure uses the double-gate architecture with III–V compound semiconductors at the channel and an N^+^-doped pocket at the junction between the source and channel regions. The drain and source regions are realized with GaSb material. HfO_2_ was used as high-*k* dielectric material, and an interfacial SiO_2_ dielectric layer was created between the high-*k* dielectric material and the body channel. The pocked region was implemented between the channel and the source regions. Two cavities were created in the pocket region side and between the gate electrode and the body channel, as shown in Figure 16. The reported PKD TFET-based biosensor by Rashid et al. exhibits very good values of current sensitivity (4.35 × 10^8^), which is one hundred times better than the biosensor structure proposed by Devi et al. [115]. This biosensor structure offers maximum sensitivity of 1.51 × 10^9^ when the cavity is fully filled with positively charged biomolecules.

**Figure 17:**
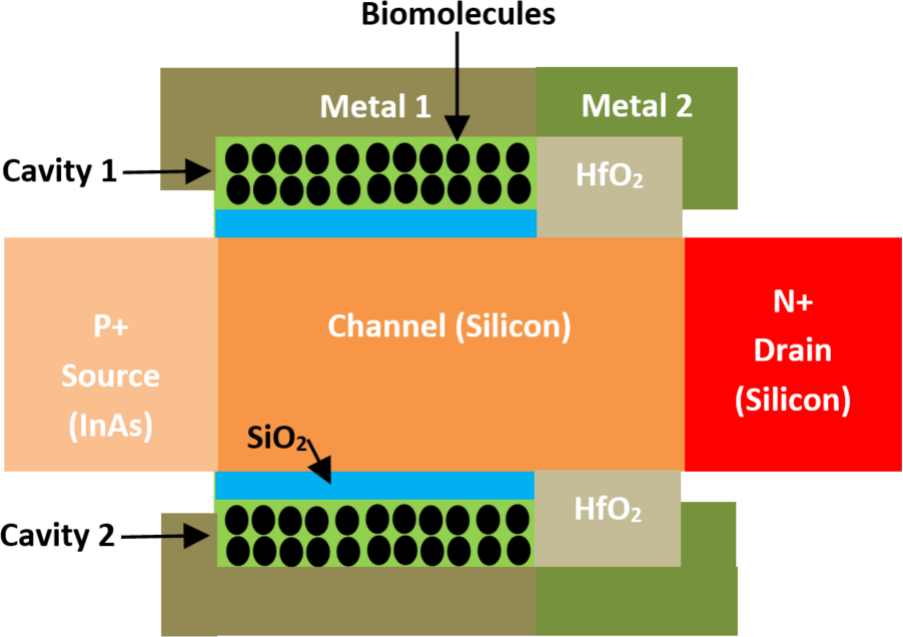
Schematic showing the structure of a GE H TFET-based biosensor.

**2.2.13 Gate-engineered hetero-structure TFET-based biosensors.** A high-performance gate-engineered heterostructure tunnel (GE H TFET)-based biosensor has been proposed by Ghosh et al. [116]. Figure 17 shows the organization of a GE H TFET-based biosensor. In this structure, dual cavities were implemented between the silicon channel and the electrode gate, each electrode gate uses two different metal materials. The first part of the electrode gate (with metal 1) is interfaced with SiO_2_ dielectric but the second part of the electrode gate (with metal 2) is interfaced with HfO_2_ high-*k* dielectric with the body channel. The source region is P^+^ doped but the drain region is N^+^ doped, as shown in Figure 17. In this context, a low bandgap material (InAs) was implemented in the source region to achieve better band-to-band tunneling of carriers. Additionally, an extended gate architecture was incorporated to attain good stability of the biomolecules at the nanocavity, significantly improving on-current sensitivity (7.96 × 10^9^) compared to available FET-based biosensors and existing tunnel FET-biosensor structures, such as double-gate TFET and Ge-source dielectric-modulated dual-gate TFET.

**2.2.14 Staggered heterojunction vertical TFET-based biosensors.** A GaSb/Si staggered heterojunction vertical tunnel FET (SHV TFET)-based biosensor has been analytically investigated and proposed for biological applications by Khan et al. [117]. The proposed SHV TFET biosensor, with the designed nanocavity segment, offers better sensitivity (≈7.7 × 10^9^) compared to that of classical DM PNPN TFET [118] and full-gate DM TFET [54]. The SHV TFET structure exhibits very good resistance to short-channel effects (SCEs) and leakage currents, making this biosensor structure promising and an excellent candidate for various highly sensitive biomedical applications.

**Figure 18:**
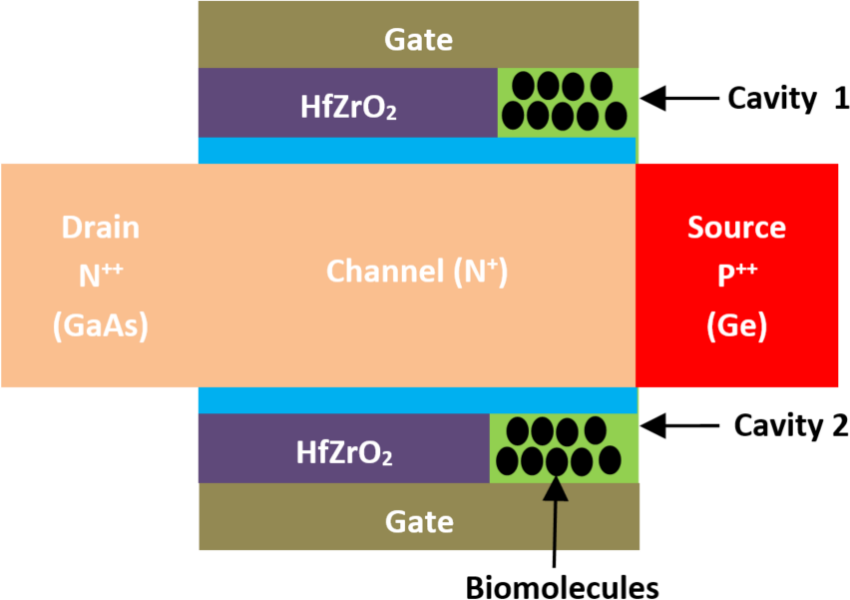
Schematic showing the structure of an NC DG TFET-based biosensor.

**2.2.15 Negative-capacitance TFET-based biosensors.** A Ge/GaAs negative-capacitance double-gate tunnel FET (NC DG) FET-based biosensor has been proposed by Paul et al. [119]. Figure 18 shows the structure of an NC DG FET-based biosensor. The main idea of this biosensor structure is the use of a ferroelectric layer between the electrode gate and the body channel targeting an ideal subthreshold slope. Moreover, this structure uses a double gate concept, a heteromaterial in the source/drain channel, and HfZrO_2_ as the ferroelectric layer. Two cavities are also created between the electrode gate and the body channel and beside the ferroelectric layer.

It has been demonstrated that the NC DG TFET-based biosensor provides high on-state current by applying lower gate voltage values, creating a steep subthreshold and therefore high current sensitivity. This type of biosensor offers high current sensitivity (9.08 × 10^12^) compared to that of classical Ge/GaAs TFET (4.04 × 10^7^) and full-gate TFET biosensors (2.5 × 10^4^) [54].

**2.2.16 Pocket-doped vertical TFET-based biosensors.** A vertical tunnel field-effect transistor (TFET)-based biosensor with N^+^-pocket doping (PKD V TFET) has been proposed by Devi et al. [115] to enhance sensitivity in FET biosensors. This structure utilizes a 30% concentration of germanium in the Si/Ge N^+^ pocket and an N-type doping concentration of 10^19^ cm^−3^.

In this case, two different gate metals with distinct work functions were designed to achieve maximum on-state current and an improved *I*_on_/*I*_off_ ratio. The fabrication process flow for the proposed biosensor structure is detailed in [120]. Sensitivity parameters were assessed by measuring the drain-current shift in relation to changes in the dielectric constant. It has been demonstrated that this type of biosensor structure exhibits a significant deviation in the drain current, making the on-state current a suitable sensing parameter. The PKD V TFET-based biosensor has been reported to demonstrate superior sensitivity compared to that of MOSFET biosensors. The proposed PKD V TFET-based biosensor exhibits enhanced sensitivity (approximately 10^6^ for a dielectric constant equal to 12) compared to that of MOSFET biosensors.

**Figure 19:**
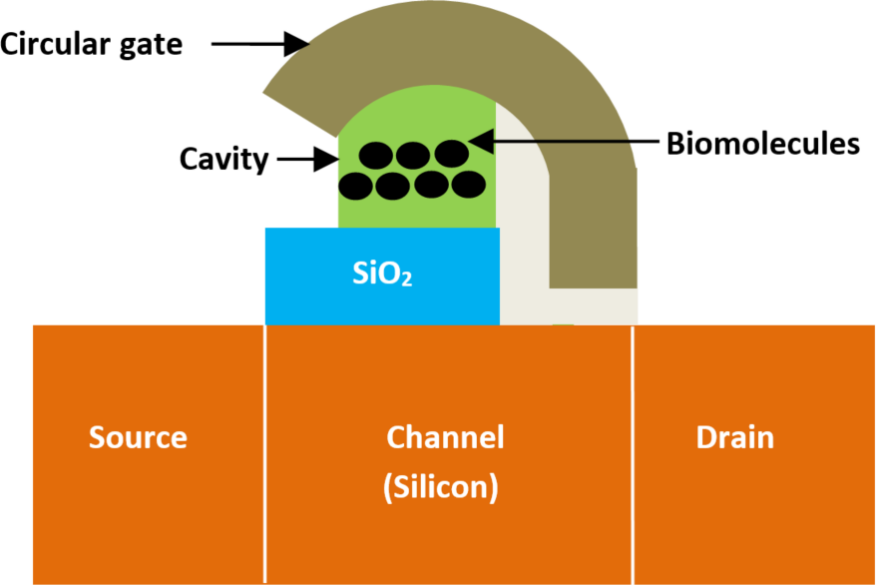
Schematic showing the structure of a CG HJ TFET-based biosensor.

**2.2.17 Circular-gate heterojunction TFET-based biosensors.** Goswami et al. [120] introduced, for the first time, a circular-gate heterojunction tunnel (CG HJ TFET)-based biosensor device. Figure 19 presents the structure of CG HJ TFET-based biosensor. The main idea of this architecture is the use of a circular electrode gate and heterojunction concept. The proposed CG HJ TFET-based biosensor provides higher sensitivity than classical heterojunction TFET-based biosensors due to the absence of a uniform gate structure. It has also been demonstrated that the sensitivity of tunnel FET biosensors is strongly dependent on the precise position of biomolecules (probe position and steric hindrance) relative to the tunnel junction. Furthermore, a maximum sensitivity of 1.31 × 10^8^ has been achieved for a fully filled nanogap CG HJ TFET-based biosensor with a dielectric constant of 12.

**2.2.18 Electrically doped junctionless TFET-based biosensors.** Chandan et al. [121] investigated electrically doped junctionless tunnel FET (ED JL TFET)-based biosensors. This structure employs a single type of doping concentration, an appropriate work function, and a polarity-gate bias. Additionally, an N^+^ heavily doped silicon layer has been implemented with two separated gates to develop P^+^ and intrinsic source regions under the control-gate and the polarity-gate electrode, each with suitable work functions and polarity bias over the silicon region. A nanogap cavity has been embedded within the control-gate dielectric and implemented by etching the control-gate dielectric region towards the polarity-gate side for sensing and biomolecule detection purposes.

It has been reported that ED JL TFET-based biosensors offer a simpler fabrication process, higher thermal budget, better sensitivity than classical devices, and good resistance to short-channel effects and drain-induced barrier-lowering impact.

**2.2.19 Pocket-doped-channel dual-cavity TFET biosensors.** Kanungo et al. [122] conducted a deep study on the impact of SiGe Source and pocket-doped-channel (SiGe S PKDC) TFET-based biosensors. In this study, the fundamental physics of the Ge composition difference in the source zone and doping density change in the N^+^-pocket region, from the perspective of biomolecule detection, is reported. It has been demonstrated that SiGe source TFET has a significant advantage over N^+^-pocket TFET biosensors in terms of subthreshold current and sensitivity.

**Figure 20:**
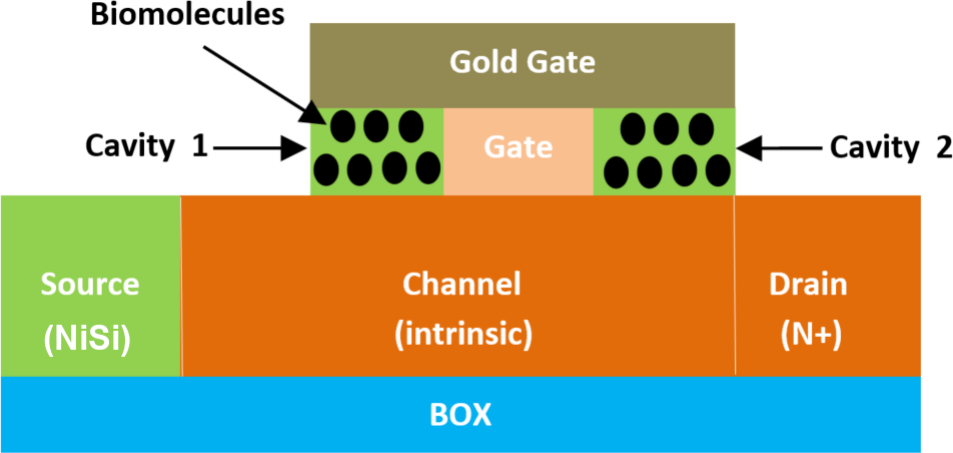
Schematic showing the structure of an ST SII FET-based biosensor.

**2.2.20 Schottky tunneling source impact-ionization-based biosensors.** Singh et al. [123] reported a concept of a nanogap-embedded Schottky tunneling source impact-ionization (ST SII) FET-based biosensor with highly sensitive detection of different neutral and charged biomolecules. Figure 20 shows the architecture of ST SII FET-based biosensors. In this structure, dual cavities were created between the body channel and an electrode gate with gold for better sensitivity. These two gates are also separated with another electrode gate positioned in the middle of the device. The silicon channel is intrinsic, the drain is N^+^ doped, and the source uses NiSi to create the Schottky tunneling structure. Furthermore, the impact-ionization effect dominates, whereas for classical DM-FETs, the influence of biomolecule charges unfavorably affects the biosensing action. It has been also indicated that ST SII FET-based biosensors offer lower voltage operation and excellent scalability, which improves reliability as a hot electron impact is significantly reduced at lower operating bias voltages. Hence, it offers higher power efficiency and reliability.

**2.2.21 Overlap gate-on-drain TFET-based biosensors.** An overlap gate-on-drain (OGOD) TFET-based biosensor has been reported by Abdi et al. [63]. This structure uses a dual nanogap, double-gate concept, and HfO_2_ as a high-*k* dielectric material between the body channel and electrode gates. The source, channel, and drain regions have been created using P^+^-, P^−^-, and N^+^-doped silicon, respectively.

In this case, the sensing parameter is linked to the modification in the ambipolar current of the biosensor with a diverse dielectric constant for the detected biomolecule. It has been indicated that OGOD TFET-based biosensors offer high sensitivity values (approximately 10^10^) and low leakage current compared to those of classical FET-based biosensors.

**2.2.22 T-shape channel TFET-based biosensors.** Shaw et al. [38] designed, for the first time, a split-gate T-shaped channel tunnel (SG TSC) TFET-based biosensor. In this structure, the middle part of the gate oxide is etched away to form a cavity in between the gate oxides on either side [95]. The suggested device combines the tunnel concept with the drain pocket. Therefore, a detailed study of the device optimization and performance of the split-gate T-shaped channel DM dual-gate TFET with a drain pocket has been performed. It has been indicated that for a dielectric constant equal to 25, the maximum value of sensitivity is 6.70 × 10^7^, and the *I*_on_/*I*_off_ ratio is almost equal to 1.44 × 10^11^. The SG TSC TFET-based biosensor outperforms various recently proposed works. The suppression of ambipolarity is also achieved by using the drain pocket concept. It has been demonstrated that SG TSC TFET-based biosensors offer the lowest value of the subthreshold slope (33 mV/Dec) and a larger *I*_on_/*I*_off_ ratio (36 times) compared to the maximum value reported by Vanlalawmpuia et al. [124]. This biosensor structure could be considered for various biosensing applications.

### 3 Comparison between emerging FET-based biosensors

**Figure 21:**
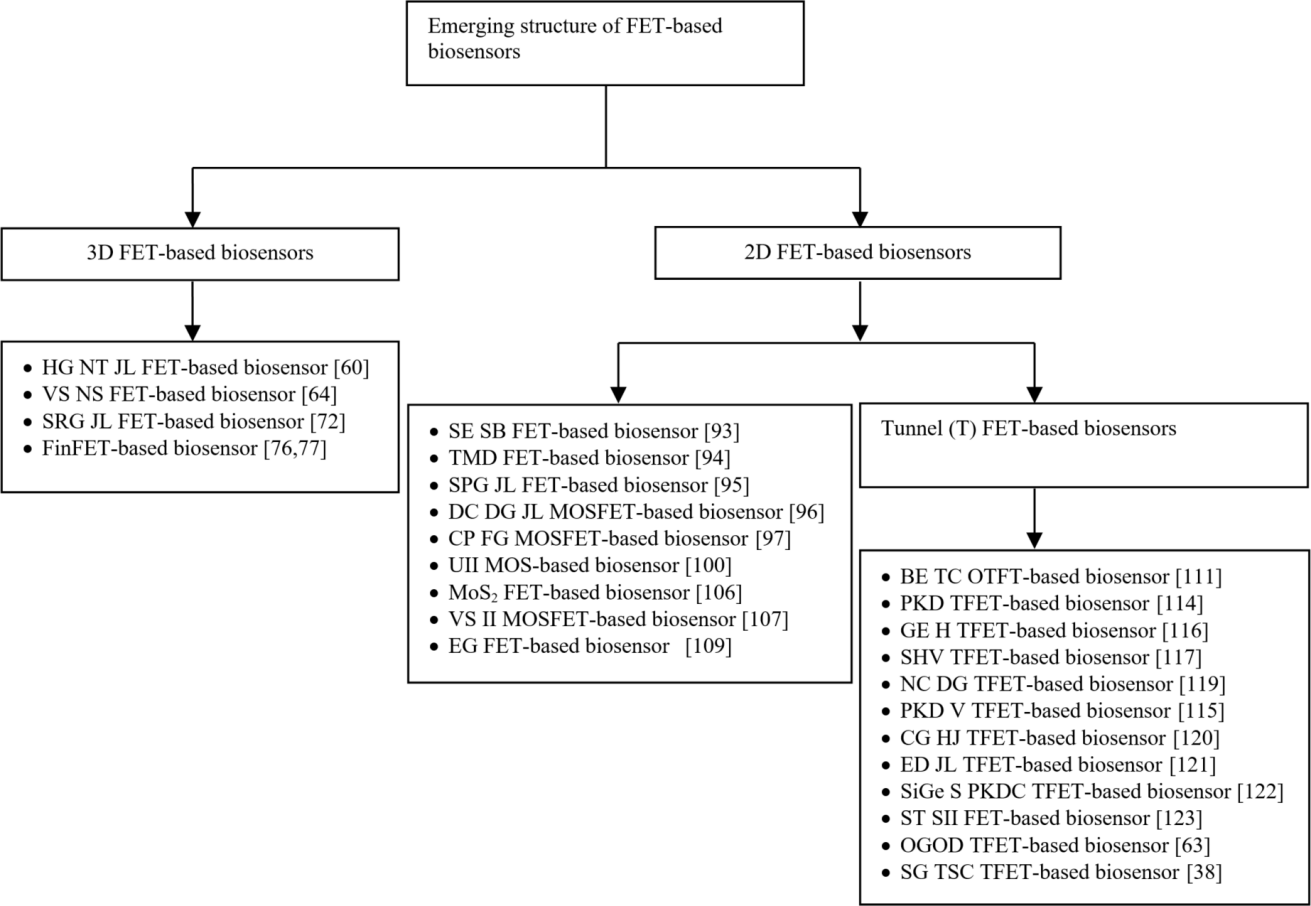
Review on emerging three-dimensional and two-dimensional FET-based biosensors.

Various research works on FET-based biosensor structures have been reported in recent years, as discussed in sections 2.1 and 2.2. In this context, FET-based biosensors are classified into two main families: three-dimensional (3D) FET-based biosensors and two-dimensional (2D) FET-based biosensors, as illustrated in Figure 21.

A detailed mapping of sensitivity parameters, along with various dielectric constants (*k*) for main FET biosensors, is presented to demonstrate the status of such biosensors, as shown in Table 1. Traditional MOSFET-based biosensors exhibit the lowest current sensitivity due to their planar structure, conventional semiconductor materials, and typical carrier transport, as observed in FET-biosensors, such as DC DG JL MOSFET-based [96] and SE SB FET-based biosensors [93].

**Table 1:** Comparison between different types of FET-based biosensors in terms of current sensitivity.

Structure	Current sensitivity	Reference

HG NT JL FET-based biosensor (*L*_cap_ = 100 nm, *L*_g_ = 100 nm, *H*_cap_ = 150 nm, *k* = 3.64)	3.00 × 10^13^	[60]
PKD TFET-based biosensor (*L*_cap_ = 15 nm, *L*_g_ = 50 nm, *H*_cap_ = 1.5 nm, *k* = 12)	1.514 × 10^9^	[114]
CS NT JL TFET-based biosensor (*L*_gap_ = 100 nm, *L*_g_ = 50 nm, *k* = 8, 50% filled)	1.9 × 10^2^	[62]
GUC (gate underlap channel) DG MOSFET-based biosensor (*L*_cap_ = 100 nm, *L*_g_ = 400 nm, *H*_cap_ = 10 nm, *k* = 10)	21.3	[128]
DM TFET-based biosensor (*L*_gap_ = 10 nm, 50 nm, *L*_gate_ = 20 nm and 11 nm, *k* = 8)	49.98	[127]
SRG JL FET-based biosensor (*L*_cap_ = 35 nm, *L*_g_ = 50 nm, *H*_cap_ = 4 nm, *k* = 1.54)	3.5 × 10^3^	[72]
SP (source pocked) DM TFET-based biosensor ( *L*_gap_ = 20 nm, *L*_ch_ = 42 nm, *k* = 2.1)	40	[126]
DM TFET-based biosensor (*L*_gap_ = 20 nm, *L*_ch_ = 42 nm, *k* = 2.1)	10	[126]
FinFET-based biosensor (*L*_cap_ = 8 nm, *L*_g_ = 10 nm, *H*_cap_ = 5 nm, *k* = 8)	1.55	[76]
DM TFET-based biosensor (*L*_gav_ = 90 nm, *L*_g_ = ≈90 nm, *k* = 12)	6.95 × 10^12^	[69]
DM PNPN TFET-based biosensor (*L*_gap_ = 20 nm, *L*_ch_ = 42 nm, *k* = 2.1)	4.11 × 10^3^	[118]
UII MOS-based biosensor (*L*_gap_ = 63 nm, *L*_g_ = 90 nm, *k* = 80)	10^7^	[100]
InAs DM NT-TFET-based biosensor (*L*_gap_ = 50 nm, *L*_g_ = 50 nm, *k* = 10)	8 × 10^5^	[129]
DC DG JL MOSFET-based biosensor (*L*_gap_ = 35 nm, *H*_cap_ = 10, *L*_g_ = 100 nm, *k* = 12)	1.02	[96]
NC DG TFET-based biosensor (*t*_Ferro_ = 5, nm, *L*_g_ = 20 nm, *k* = 12)	9.07 × 10^13^	[119]
SE SB FET-based biosensor (*L*_cap_ = 35 nm, *H*_cap_ = 5 nm, *k* = 10)	3.25	[93]
CG HJ TFET-based biosensor (*L*_cap_ = 25 nm, *L*_g_ = 40 nm, *H*_cap_ = 11 nm, *k* = 12)	1.31 × 10^8^	[120]
OGOD TFET-based biosensor (*L*_cap_ = 25 nm, *L*_g_ = 50 nm, *H*_cap_ = 9 nm, *k* = 10)	1.00 × 10^10^	[63]
GE H TFET-based biosensor (*L*_cap_ = 30 nm, *L*_g_ = 40 nm, *H*_cap_ = 10 nm, *k* = 12)	7.95 × 10^9^	[116]

On the other hand, 2D TFET-based biosensors showed improved sensitivity due to differences in current injection mechanisms [125], exemplified by PKD TFET-based biosensors [114], and especially the NC DG TFET-based biosensors, which offer the best sensitivity [119] due to the incorporation of negative capacitance (NC) and the tunneling mechanism, offering ideal subthreshold swing and enhanced current injection variability than those of conventional and tunnel FET biosensors, such as SRG JL FET-based [72] and DM PNPN TFET-based biosensors [118].

Furthermore, it is observed that GE H TFET-based biosensors enhance the current sensitivity of FET-based biosensors [116] compared to other TFET-based biosensor topologies [126–127] and conventional FET-based biosensors [128] by utilizing a gate-engineered heterostructure and low bandgap materials in the source region, which facilitates efficient carrier band-to-band tunneling. Furthermore, extended gate architecture is integrated to stabilize biomolecules within the nanocavity [116], resulting in higher on-current sensitivity in GE H TFET-based biosensors than those of existing tunnel and classical FET-biosensor structures.

Additionally, 3D HG NT JL FET-based biosensors demonstrate high-current sensing capabilities due to nanotube junctionless surrounding heterogate structure combined with junctionless concept, and tunnel mechanism of carrier transport [60] in comparison with other conventional metal gate TFET-biosensors, such as core–shell junctionless TFET (CS NT JL TFET)-based biosensors [62] and PKD TFET-based biosensors [114].

In addition, the comparative study indicates that among diverse FET biosensors, PKD TFET- [114], DM CTG FET- [69], NC DG TFET- [119], CG HJ TFET- [120], OGOD TFET- [63], GE H TFET- [116], and 3D HG NT JL FET-based biosensors [60] are promising for nanoscale FET biosensor design and application, as these biosensor topologies exhibit the best current sensitivity and enhanced performances. The PKD TFET-based biosensor uses III–V compound semiconductors in the body channel and N^+^-doped pockets at the source–channel junction [114], enhancing sensitivity for the detection of biomolecules. The CG HJ TFET-based biosensor utilizes heterojunctions and a circular gate shape, offering higher sensitivity than conventional TFET-based biosensors due to its nonuniform gate structure [120]. The OGOD TFET-based biosensor features an overlap gate on the drain, a dual nanogap, a double-gate design, and HfO_2_ as a high-*k* dielectric material between the body channel and electrode gates. The source, channel, and drain regions are constructed using P^+^-, P^−^-, and N^+^-doped silicon, respectively [63]. Sensing parameters are linked to modifications in the ambipolar current of the biosensor with diverse dielectric constants for detected biomolecules, resulting in high sensitivity values and low leakage currents compared to other TFET-based biosensors [120,129]. The finFET structure [130–131] with negative capacitance heterogate structure can also play a crucial role in designing a high-sensitive FET-based biosensor.

### 4 Summary and future research works

FET-based biosensors have been designed and developed to achieve higher performance and improved sensitivity in detecting various types of species, such as viruses, cancer cells, proteins, DNA, glucose, and nucleic acids. The latest emerging 3D and 2D FET-based biosensor architectures are summarized in Figure 21. A comparison of main FET-based biosensors with respect to current sensitivity is presented in Table 1. The 3D heterogate nanotube junctionless FET-based biosensor and 2D negative-capacitance (NC) tunnel FET-based biosensor structures are excellent variants for designing highly sensitive FET biosensors.

However, there are still several possibilities that can be recommended for future work, such as implementation of artificial intelligence (AI) and machine learning (ML) algorithms for 3D and 2D FET-based biosensors. In this regard, the ML-based neural network (NN) could be used to predict device parameters and performance of 3D and 2D FET-based biosensors. NN and ML could be employed to predict and study the scalability of FET biosensors. Also, they could be applied to the fabrication process of these types of biosensors. Therefore, AI algorithms and methods could be applied to expedite the development of new FET biosensor technologies. NN and ML could also be used for the development and design of 3D negative capacitance tunnel FET-based biosensors, design and implementation of gate-engineered heterostructure tunnel 3D gate-all-around FET-based biosensors, and to study temperature effects on 2D and 3D FET-based biosensors.

## Conclusion

This review introduces the significant evolution of FET-based biosensors. Various novel architectures of FET-based biosensors are summarized, providing valuable insights for enhancing sensitivity and designing nanoscale biosensors with improved performance across different applications. The discussion covers diverse topology concepts, including HG NT JL FET-, VS NS FET-, SRG JL FET-, FinFET-, SE SB FET-, TMD FET-, SPG JL FET-, DC DG JL MOSFET-, CP FG MOSFET-, BE TC OTFT-, PKD TFET-, GE H TFET-, and NC DG TFET-based biosensors. The 3D HG NT JL FET-based biosensor stands out for its superior sensitivity and overall performance compared to other 3D FET biosensors. Tunnel FET-based biosensors exhibit higher sensitivity owing to differences in current injection mechanisms, while the incorporation of the negative-capacitance concept enhances the primary performance of the biosensor. Furthermore, the synergy of 3D FET with the tunnel negative-capacitance concept emerges as a promising alternative for designing highly sensitive FET biosensors.

## Data Availability

Data sharing is not applicable as no new data was generated or analyzed in this study.
